# HiCP2GAN: a plug and play foundation model-based GAN for Hi-C enhancement

**DOI:** 10.1093/bib/bbag610

**Published:** 2026-11-02

**Authors:** Samuel Olowofila, Oluwatosin Oluwadare

**Affiliations:** Department of Computer Science, University of Colorado at Colorado Springs, 1420 Austin Bluffs Pkwy, Colorado Springs, CO 80918, United States; Department of Computer Science, University of Colorado at Colorado Springs, 1420 Austin Bluffs Pkwy, Colorado Springs, CO 80918, United States; Department of Computer Science and Engineering, University of North Texas, 1155 Union Circle, Denton, TX 76203, United States

**Keywords:** Hi-C, foundation models, resolution enhancement, 3D chromosome reconstruction, vision transformer, generative adversarial network

## Abstract

The 3D organization of chromatin shapes gene regulation and cellular function, and Hi-C has become the primary technique for mapping chromatin interactions genome-wide. However, high-resolution data remain costly and scarce, leaving many studies with sparse contact maps that limit downstream analysis. Deep learning methods, especially generative adversarial networks (GANs), have shown promise for enhancing low-resolution Hi-C data, but most existing GAN-based approaches rely on custom discriminators trained from scratch, which can yield unstable training and limited generalization. Hi-C foundation models pretrained on large-scale data capture rich, transferable representations of chromatin structure, yet their use as discriminators within adversarial enhancement frameworks had not been explored. We introduce HiCP2GAN, a plug-and-play GAN that employs a pretrained Vision Transformer-based Hi-C foundation model as its discriminator. The discriminator was pretrained on 118 million Hi-C patches across diverse species and cell types, providing biologically meaningful gradients for adversarial supervision. The framework is generator-agnostic: any compatible Hi-C resolution enhancement architecture can serve as the generator, enabling fair comparison across methods. The encoder phase of the foundation model was adapted as a discriminator backbone, finetuning the first few layers from the input while freezing the deeper transformer layers to preserve pretrained knowledge while allowing task-specific adaptation. Experiments across various cell lines show that HiCP2GAN outperforms traditional GAN-based methods in most settings and improves the reconstruction quality and biological reproducibility of existing Hi-C enhancement models in most evaluated cases. HiCP2GAN is publicly available at https://github.com/OluwadareLab/HiCP2GAN.

## Introduction

The spatial organization of chromatin in the cell nucleus plays a cardinal role in modeling the gene regulatory landscape. Hi-C, the prime high-throughput chromosome conformation capture technique for genome-wide assessment of chromatin interaction frequencies (IF) provides critical insights into higher-order chromatin structures, loops, and topologically associating domains (TADs) [1]. However, the required high-resolution (HR) data across cell types and experimental conditions are inherently constrained by sequencing depth, experimental cost, and data noise.

A growing body of research has focused on mitigating these limitations using computational enhancement techniques. Early approaches leveraged statistical modeling [2], while later methods explored deep learning frameworks including convolutional neural networks (CNNs), such as HiCNN1 [3], HiCPlus [4], HiCNN2 [5], HiCARN1 [6], SRHiC [7], and HiCPlus [4]; generative adversarial networks (GANs) [8] inclusive of HiCSR [9], HiCARN2 [6], HiCGAN [10], DeepHiC [11], and EnHIC [12], and Attention-based mechanisms such as DiCARN [13, 14].

In spite of these advances, most Hi-C resolution enhancement methods still suffer from constraints, including monolithic architectures, tightly coupled generator designs, resolution hallucination and assumptions, normalization strategies, and loss function mismatching. As a result, direct comparison across methods is confounded by incompatible workflows, differing output ranges, and inconsistent evaluation protocols. Also, the majority of GAN-based approaches exhibit shallow convolutional discriminators that essentially capture local feature data, resulting in limited abilities to capture long-range chromatin dependencies that are crucial to 3D genome organization and analyses.

Across the GAN-based methods proposed for Hi-C enhancement including hicGAN [10], DeepHiC [11], HiCSR [9], HiCARN2 [6], and EnHiC [12], the discriminators are typically shallow CNNs trained from scratch, limiting their ability to encode long-range chromatin topology (Table 1).

**Table 1:** Overview of GAN-based Hi-C enhancement methods, showing the discriminator architecture across methods.

Method	Discriminator	Foundation model
hicGAN	CNN	No
DeepHiC	CNN	No
HiCSR	CNN	No
HiCARN2	CNN	No
EnHiC	CNN	No
HiCP2GAN (Ours)	ViT (pretrained)	Yes

In recent computational evolution, foundation models [15, 16], typically pretrained on very large domain-centered datasets and have demonstrated remarkable success across various domain-specific tasks ranging from computer vision to natural language processing, and more importantly, biological modalities. The Hi-C analyses domain has recently recorded the encoding of rich chromatin structure representation when using transformer-based architectures pretrained on diverse contact maps, demonstrating the global interaction patterns adaptable for varying downstream exploration [17].

In this study, we introduce HiCP2GAN, a novel generator-agnostic generative adversarial method for Hi-C reconstruction that integrates a pretrained Hi-C foundation model as its backbone discriminator. HiCP2GAN tackles the Hi-C resolution enhancement problem with foundation-supervised adversarial learning, where various generator architectures are evaluated and optimized under a unified, semantically nuanced discriminator.

One key idea underlying HiCP2GAN is the decoupling of the generator construction from its adversarial supervision. By enforcing a standardized input–output interface, consistent numerical representation, and shared discriminator architecture, HiCP2GAN enables fair, controlled comparison across heterogeneous enhancement methods. The adopted foundation-based discriminator [17] uses self-attention [18] to capture long-range chromatin interactions, thus providing adversarial feedback that adequately reflects biologically meaningful structural properties instead of purely local signal fidelity.

We demonstrate that adversarial training with a transformer discriminator improves Hi-C reconstruction in most settings, while revealing inherent architectural trade-offs as well as strengths within the various generator models. HiCP2GAN could now serve as both a benchmarking framework and a generalizable training scaffold for future Hi-C enhancement methods.

## Materials and methods

### HiCP2GAN framework overview

HiCP2GAN is an adversarial enhancement framework designed to enhance the spatial fidelity of low-resolution (LR) Hi-C contact matrices while remaining agnostic to the underlying generator architecture. Unlike prior Hi-C enhancement models that tightly couple architectural design, resolution assumptions, and loss formulations into a single model, HiCP2GAN decouples generator construction from discriminator supervision through the integration of a foundation-level representation learner.

The framework consists of three principal components: (i) a plug-and-play generator module responsible for super-resolving low-resolution Hi-C patches, (ii) a transformer-based discriminator derived from the pretrained HiCFoundation model [17], and (iii) a standardized interface that enforces consistent dimensionality and numerical representation across heterogeneous generators.

Given a low-resolution Hi-C contact matrix (*40 × 40* patches), the generator produces a super-resolved prediction that is normalized into a biologically valid IF range and evaluated by the discriminator. The discriminator processes both generated and real high-resolution matrices to produce adversarial realism scores [8] while also extracting intermediate feature representations used for feature-matching regularization.

The discriminator backbone uses a ViT architecture [19] pretrained on large-scale Hi-C contact maps. This design enables the adversarial classifier to encode long-range chromatin interactions, multi-scale interaction patterns, and higher-order structural dependencies that are difficult to capture using conventional convolutional discriminators.

The overall architecture of HiCP2GAN is illustrated in Fig. 1.

**Figure 1:**
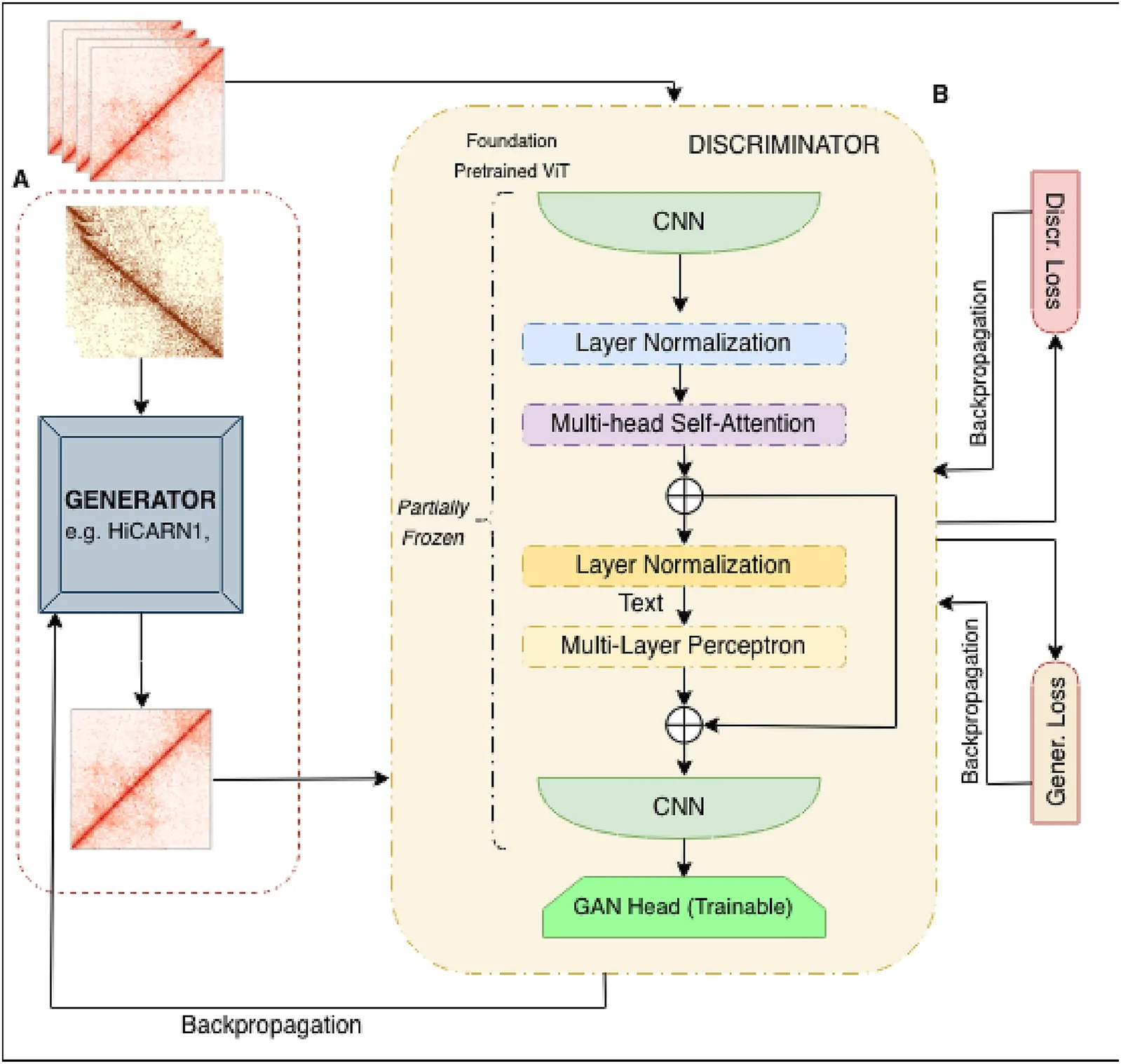
Architecture of the HiCP2GAN plug-and-play adversarial framework for Hi-C resolution enhancement. (A) *Generator*: A generator-agnostic enhancement module (e.g. HiCARN1, DiCARN, or other compatible architectures) takes low-resolution Hi-C contact matrices as input and produces enhanced Hi-C maps, enabling seamless integration of diverse enhancement models within a unified adversarial framework. (B) *HiCFoundation Discriminator*: A pretrained transformer-based discriminator initialized from HiCFoundation provides structural priors for chromatin interaction modeling. The discriminator consists of convolutional feature extractors, multi-head self-attention blocks, and feed-forward layers followed by a lightweight GAN head. The deeper transformer layers are frozen to preserve pretrained representations, while the earlier layers (closer to the input) and GAN head are finetuned during adversarial training. Adversarial and feature-matching losses guide generator optimization, enabling resolution enhancement informed by both local convolutional patterns and global chromatin interaction structure.

### Generator-agnostic interface and data standardization

Hi-C enhancement models differ substantially in their architectural assumptions, including input resolutions, output ranges, preprocessing pipelines, and training objectives. These inconsistencies complicate direct comparison across methods and can introduce confounding factors during adversarial training.

HiCP2GAN addresses this problem through a generator-agnostic plug-and-play interface that standardizes input resolution and numerical representation while preserving the internal structure of each generator.

All generators operate on *40 × 40* LR Hi-C patches and produce enhanced outputs at the same resolution. Models originally designed for *28 × 28* inputs are integrated through deterministic resolution adaptation. Specifically, low-resolution patches are resized from *40 × 40* to *28 × 28* using area-based interpolation prior to inference and restored to the original resolution using nearest-neighbor interpolation after enhancement. No additional learnable upsampling layers are introduced, ensuring that generators are not granted extra trainable capacity beyond their original design.

To maintain numerical consistency across models, all generator outputs are normalized to the interval *[0,1]*. Outputs produced using *\tanh * activation (e.g. HiCSR) are linearly rescaled to this range, whereas outputs already operating within the target interval remain unchanged. All predictions are subsequently clamped to ensure numerical stability. Ground-truth high-resolution matrices are likewise clamped prior to computing reconstruction losses and evaluation metrics.

This standardization guarantees compatibility with reconstruction losses, perceptual similarity metrics, and biological reproducibility measures such as GenomeDISCO [20], enabling unbiased benchmarking across heterogeneous generator architectures.

### Foundation-based discriminator architecture

The HiCP2GAN discriminator is derived from HiCFoundation [17], a ViT pretrained on large-scale Hi-C datasets. Input Hi-C patches are first projected to three channels through a lightweight convolutional adapter before being processed by the transformer encoder.

To adapt the pretrained encoder for adversarial learning, a lightweight fully connected classification head is appended to produce a scalar realism score indicating whether an input matrix is real or generated. During adversarial training, the deeper transformer blocks are frozen to preserve pretrained chromatin interaction representations, while the earlier blocks (towards the input end) and the adversarial head are both finetuned. The optimal finetuning depth is determined experimentally (Section Discriminator freezing and model validation).

The discriminator serves a dual role during training. In addition to adversarial classification, intermediate transformer representations are exposed for feature-matching regularization. These features enable the generator to align its outputs with structural representations learned by the pretrained foundation model, encouraging consistency with biologically meaningful chromatin interaction patterns.

### Adversarial objective and optimization

HiCP2GAN is trained using a composite adversarial objective that balances structural realism, signal fidelity, and representation-level consistency across embeddings. Let $X_{\textrm{LR}}$ denotes a low-resolution Hi-C patch, $X_{\textrm{HR}}$ the corresponding high-resolution matrix, and $\tilde{X}_{\textrm{SR}} = G(X_{\textrm{LR}})$ the normalized generator output.

The discriminator *D(· )* is trained to distinguish real high-resolution matrices from generated samples using binary cross-entropy with logits:


(1) \begin{align*}& \mathcal{L}_{D} = \mathbb{E}_{X_{\textrm{HR}}} \left[ \ell_{\mathrm{BCE}}(D(X_{\textrm{HR}}), 1) \right] + \mathbb{E}_{X_{\textrm{LR}}} \left[ \ell_{\mathrm{BCE}}(D(\tilde{X}_{\textrm{SR}}), 0) \right],\end{align*}

where $\ell _{\mathrm{BCE}}(\cdot ,\cdot )$ denotes the binary cross-entropy loss with logits.

The generator adversarial loss encourages generated samples to be classified as real:


(2) \begin{align*}& \mathcal{L}_{\mathrm{adv}} = \mathbb{E}_{X_{\textrm{LR}}} \left[ \ell_{\mathrm{BCE}}(D(\tilde{X}_{\textrm{SR}}), 1) \right].\end{align*}

To preserve contact-intensity fidelity, we employ an $\ell _{1}$ reconstruction loss:


(3) \begin{align*}& \mathcal{L}_{\mathrm{recon}} = \left\lVert \tilde{X}_{\textrm{SR}} - X_{\textrm{HR}} \right\rVert_{1}.\end{align*}

To align generated and real samples in the discriminator representation space, we further apply a feature-matching loss. Let $\phi (\cdot )$ denotes the pooled transformer representation extracted from the discriminator. The feature-matching loss is defined as:


(4) \begin{align*}& \mathcal{L}_{\mathrm{FM}} = \left\lVert \phi(\tilde{X}_{\textrm{SR}}) - \phi(X_{\textrm{HR}}) \right\rVert_{1}.\end{align*}

The total generator objective is:


(5) \begin{align*}& \mathcal{L}_{G} = \lambda_{\mathrm{adv}} \mathcal{L}_{\mathrm{adv}} + \lambda_{L1} \mathcal{L}_{\mathrm{recon}} + \lambda_{\mathrm{FM}} \mathcal{L}_{\mathrm{FM}},\end{align*}

where $\lambda _{\mathrm{adv}}$, $\lambda _{L1}$, and $\lambda _{\mathrm{FM}}$ are scalar weighting coefficients fixed across all experiments.

Training proceeds via alternating updates of the discriminator and generator. For each minibatch, the discriminator is first updated using real and generated samples, followed by a generator update using the composite objective above. Both networks are optimized using the Adam optimizer with identical learning rates and momentum parameters.

Automatic mixed-precision (AMP) training is employed to improve computational efficiency while maintaining numerical stability. Gradient clipping is applied during both generator and discriminator updates to mitigate gradient explosions during early training stages.

## Results

### Experimental setup

#### Hi-C data description and preprocessing

All experiments were conducted on genome-wide Hi-C contact matrices generated at a fixed resolution of 10 kb across all evaluated cell lines. The dataset includes four widely used human cell lines and one mouse cell line originating from the study of Rao *et al*. [21]: GM12878 (lymphoblastoid cells), K562 (leukemia cells), HMEC (mammary epithelial cells), NHEK (epidermal keratinocytes), and CH12-LX (mouse B-cell lymphoma) cell line.

Of the five cell lines evaluated in this study, only HMEC and NHEK were included in the HiCFoundation pretraining corpus, whereas GM12878, K562, and CH12-LX (listed as CH12) were assigned to the test split and excluded from foundation-model pretraining. The comprehensive accession list for the datasets used to develop and evaluate HiCFoundation is provided in the supplementary material of Wang *et al*. [17]; the accessions relevant to the present study are summarized in Supplementary Table S1. In our study, task-specific training of HiCP2GAN uses GM12878 only; therefore, K562, HMEC, NHEK, and CH12-LX are held out from HiCP2GAN training. The discriminator is initialized using the pretrained HiCFoundation backbone and fine-tuned on GM12878 data downsampled at *1/16* and *1/64* for Hi-C resolution enhancement. Thus, K562 and CH12-LX are held out from both HiCFoundation pretraining and HiCP2GAN training, supporting evaluation of cross-cell-line generalization and, in the case of CH12-LX, cross-species generalization.

To simulate shallow-sequenced Hi-C data, we uniformly downsampled the *in-situ* high-resolution contact maps using a ratio of 16 and 64 for a dual LR-variability study. This downsampling protocol was applied consistently across training, fine-tuning, validation, and testing datasets for all corresponding procedures.

We emphasize that both the input and target maps are represented at the same 10 kb genomic bin size; the low-resolution input is produced by reducing effective sequencing depth, which increases sparsity and noise, rather than by coarsening the genomic bin size. The task is therefore one of low-depth or shallow sequenced Hi-C enhancement rather than spatial super-resolution in the image sense, and we use this terminology throughout.

To prevent information leakage across genomic loci, we adopt a strict chromosome-disjoint split strategy across train, validation, and test datasets as follows:

- Training chromosomes: chr1, chr3, chr5, chr7, chr8, chr9, chr11, chr13, chr15, chr17, chr18, chr19, chr21, and chr22
- Validation chromosomes: chr2, chr6, chr10, and chr12
- Test chromosomes: chr4, chr14, chr16, and chr20

#### Training

All models were trained for a fixed number of epochs with a batch size of 64, and AMP [22] was used to improve computational efficiency.

For our adversarial framework, the generator was optimized using a weighted combination of three loss components: (i) adversarial loss [23], (ii) pixel-wise $\ell _{1}$ reconstruction loss [24], and (iii) feature-matching loss computed from intermediate discriminator representations [25]. Gradient clipping was applied to both generator and discriminator updates to stabilize training.

All experiments were executed on NVIDIA TITAN RTX GPUs, while the core training and parameter optimization settings for HiCP2GAN experiments are provided in Supplementary Table S2 and Fig. S2.

#### Validation protocol and metrics

We adopted the PSNR [26] computation metric, popularly used for measuring reconstruction fidelity for our validation benchmarking, while the Structural Similarity Index (SSIM) [27], capturing perceptual and structural consistency, and GenomeDISCO [20] for assessing biological reproducibility of chromatin interaction patterns, are reported in the test scenarios.

### Discriminator freezing and model validation

We conducted a discriminator finetuning-depth ablation to characterize how the pretrained HiCFoundation backbone responds to varying degrees of task-specific adaptation. Rather than identifying a single optimal configuration, this analysis was designed to assess the transferability of pretrained chromatin representations under adversarial supervision.

#### Phase I: Discriminator finetuning depth ablation

Partial layer freezing is a transfer-learning technique that preserves general features learned during large-scale pretraining while enabling task-specific adaptation [28]. Howard and Ruder [29] further demonstrated that gradual unfreezing can improve stability while preventing catastrophic forgetting. We adopt a complementary strategy: finetuning early transformer layers while freezing deeper ones, preserving higher-level pretrained representations while allowing input-proximal layers to adapt to the enhancement task. We varied the number of finetuned layers incrementally from 0 (fully frozen) to 24 (fully finetuned).

As shown in Supplementary Table S3 and Fig. 2, remarkable performance is recorded across the full range of finetuning depths, with the configurations achieving near-identical validation PSNR. Figure 2 presents the epoch-wise validation PSNR plots for each finetuning depth. Notably, the fully frozen discriminator (0 finetuned layers) performs comparably to fully or partially finetuned variants, suggesting that the HiCFoundation backbone transfers rich chromatin interaction representations directly to adversarial Hi-C enhancement while preserving strong performance across varying adaptation settings.

**Figure 2:**
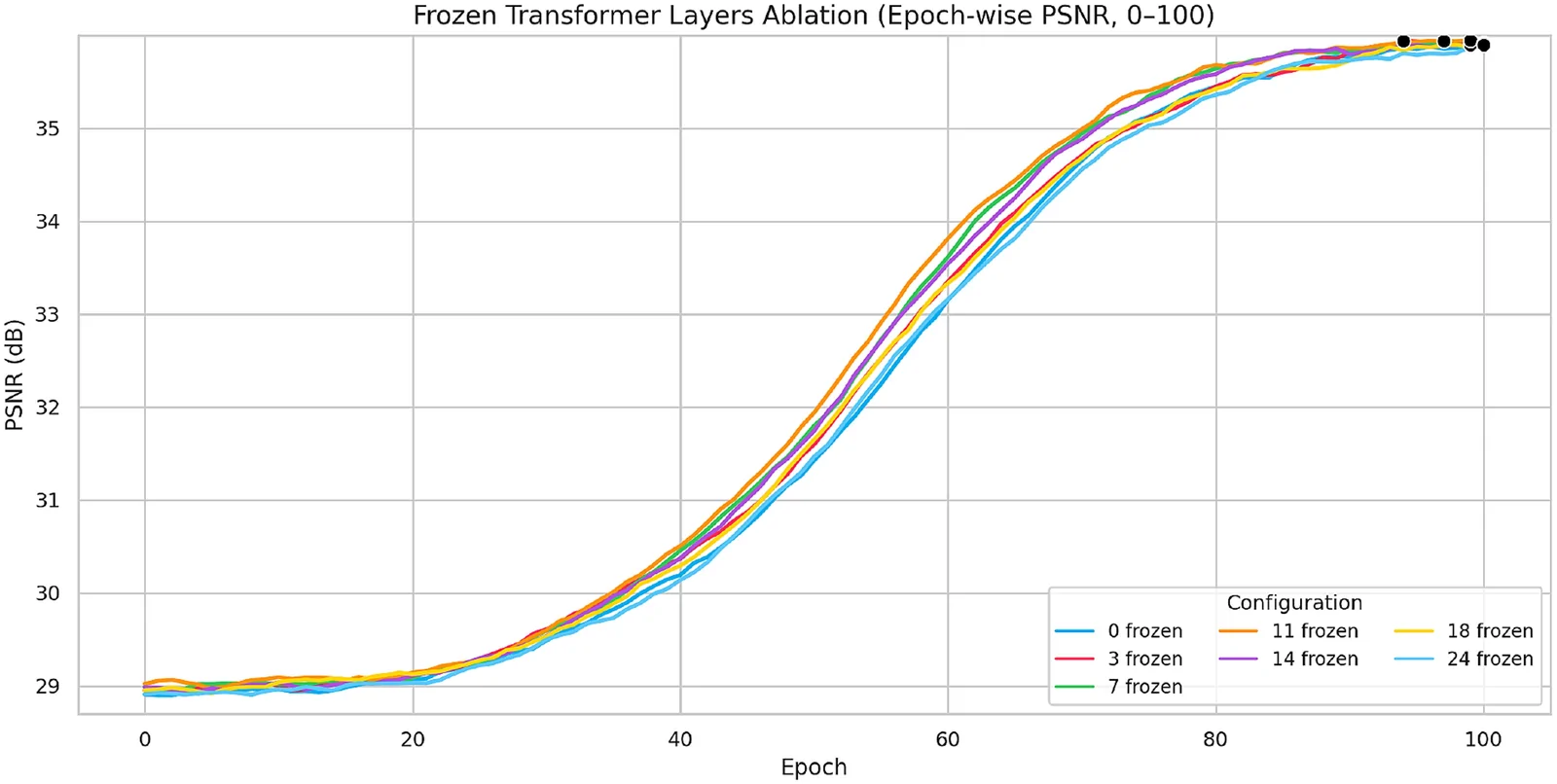
Layer finetuning validation of the HiCFoundation discriminator (100 epochs). Epoch-wise validation PSNR plots for HiCP2GAN trained with varying numbers of finetuned transformer layers (from the input side); deeper layers remain frozen. This was used to select the discriminator configuration adopted in our subsequent experiments.

This finding strengthens the central motivation of HiCP2GAN: that a foundation-pretrained discriminator provides an inherently richer supervisory signal than a convolutional discriminator trained from scratch, regardless of finetuning configuration. Given this plateau, the objective of discriminator configuration selection shifts from maximizing reconstruction fidelity to identifying the most parameter-efficient operating point within the stable performance region. The best-performing configurations clustered between 10 and 17 finetuned layers, which we carry forward into Phase II for efficiency-targeted analysis.

Extended training (500 epochs) yields modest gains (Supplementary Table S4) but the additional compute overhead is not justified; hence, we adopt 100 epochs for all experiments.

#### Phase II: Depth efficiency-targeted discriminator fine-tuning

To further refine the fidelity capability recorded in phase I, we conducted a second-phase experimentation solely focusing on this high-performing configuration cluster. In this phase, each configuration within the range of 10–17 blocks was trained independently with all selected layers fully fine-tuned while the deeper layers were completely truncated. This phase was inspired by the need to identify the most stable and efficient operating point within the optimal depth region identified during Phase I.

Table 2 reports the resulting PSNR values averaged across runs with their corresponding statistical variability. The results confirm that performance remains remarkably stable across the cluster, with minimal variance across configurations. However, the layout involving fine-tuning the first 11 transformer layers provided the best trade-off between reconstruction quality and training efficiency, achieving the highest average PSNR and best standard deviation. Hence, the adoption as the preferred HiCP2GAN discriminator configuration.

**Table 2:** PSNR report of phase II efficiency-targeted finetuning of GM12878 using HiCARN1 generator, with independently trained configurations retaining selected layers (10-17); the best score is recorded in bold text.

Fine-tuned layers	PSNR (dB) *± * SD
10	35.93 *± * 0.003
11	**35.93 *± * 0.002**
12	35.92 *± * 0.003
13	35.93 *± * 0.003
14	35.92 *± * 0.004
15	35.92 *± * 0.003
16	35.93 *± * 0.004
17	35.92 *± * 0.003

#### Controlled ablation: isolating the discriminator contribution

The finetuning-depth study above varies how much of a *pretrained* discriminator is adapted, but does not by itself separate the contribution of pretraining from that of discriminator architecture, capacity, and the auxiliary loss terms. To isolate these factors, we conducted a controlled ablation in which the generator (HiCARN1), optimization schedule, training data, and evaluation protocol were held fixed, while only the discriminator or active loss terms were varied. We compared seven configurations: (i) a reconstruction-only (recon_only) generator using $\ell _{1}$ loss without a discriminator; (ii) a conventional convolutional discriminator trained from scratch (cnn_disc); (iii) a randomly initialized ViT discriminator with an architecture identical to HiCFoundation (random_vit); (iv) a finetuned HiCFoundation discriminator trained with $\ell _{1}{+}$adversarial loss (l1_adv); (v) a finetuned HiCFoundation discriminator trained with $\ell _{1}{+}$feature-matching loss (l1_fm); (vi) a frozen pretrained HiCFoundation discriminator (frozen pretrained ); and (vii) the proposed partially finetuned HiCFoundation discriminator (partial_ft). Validation results on GM12878 under *1/16* downsampling are reported in Supplementary Table S8.

Four observations follow. First, on this strong generator, the pixel-fidelity metrics are near saturation: PSNR and SSIM differ only marginally across the discriminator and loss configurations, making GenomeDISCO the principal discriminating metric. Second, the conventional convolutional discriminator trained from scratch collapsed under the same training schedule and performed far below all transformer-based variants, supporting the use of a transformer discriminator for more stable and informative supervision. Third, feature matching is the operative auxiliary loss term: adding it increases GenomeDISCO from *0.909* to *0.913*, whereas the adversarial objective alone, without feature matching, matches the reconstruction-only baseline. Fourth, the proposed partially finetuned HiCFoundation discriminator (HiCP2GAN) achieves the best result on every metric, while both pretrained discriminator variants—the frozen and partially finetuned models—outperform the randomly initialized ViT of identical architecture on GenomeDISCO. This improvement therefore reflects the benefit of pretrained representations rather than transformer architecture or model capacity alone.

We therefore interpret HiCP2GAN as a stable, generator-agnostic adversarial scaffold whose benefit on this generator is concentrated in biological reproducibility (GenomeDISCO) and arises from feature matching against a pretrained transformer discriminator, with both the foundation-model pretraining and the feature-matching term contributing. We note that on this strong generator the absolute margins are small; the practical benefit of the framework is clearest across standalone generators and downstream structural tasks (Sections “HiCP2GAN improves Hi-C resolution over standalone generators and outperforms traditional generative adversarial network-based methods on seen cell line,” “HiCP2GAN enables cross-cell and cross-species transfer with superior computational and biological fidelity,” “HiCP2GAN improves loop detection over standalone generators,” “HiCP2GAN improves topologically associating domain detection and quality,” and “HiCP2GAN improves 3D genome structure reconstruction”). This ablation is intended to establish a single configuration baseline: we fix a high-performing generator and vary one component at a time under a common schedule and initialization, so that the configurations are directly comparable with one another. We do not claim that this configuration is optimal for every generator, and the comparison is not designed to characterize variability across independent training runs; the smaller margins, in particular between the randomly initialized and the pretrained discriminators, are accordingly read as indicative rather than definitive. We scope this ablation explicitly to a single strong generator, same-cell evaluation, and *1/16* downsampling, and report full results in Supplementary Table S8.

### HiCP2GAN improves Hi-C resolution over standalone generators and outperforms traditional generative adversarial network-based methods on seen cell line

For a fair comparison, all baseline methods reported in this study, including the traditional GAN-based models, were retrained and evaluated by us under an identical protocol including the same chromosome split, downsampling procedure, normalization, and evaluation code, rather than taken from previously reported values. In addition, since the GM12878 cell line is not part of the HiCFoundation corpus, HiCP2GAN has no advantage over the other models in this analysis in terms prior data exposure. Hence, by seen cell line here, we mean our training, validation, and test chromosomes split as described in SectionHi-C data description and preprocessing is performed on the same cell line. Table 3 compares standalone generators with their HiCP2GAN adaptation. Every evaluated generator, including DiCARN, HiCARN1, HiCNN, and HiCPlus, improves when trained within the HiCP2GAN framework. The gains recorded are largely consistent across PSNR, SSIM, and GenomeDISCO, reinforcing the hypothesis that foundation-supervised adversarial learning benefits a range of architectures training and testing on the same cell line (GM12878), but mutually exclusive chromosomes.

**Table 3:** Same-cell evaluation on GM12878. Standalone generators, the respective HiCP2GAN-integrated variants, and traditional GAN-based models are compared on chromosomes 4, 14, 16, and 20 of the GM12878 cell line. Bold values indicate improvement over standalone, underline indicates the best traditional GAN-based method, and ($^{\textrm{a}}$) denotes the best-performing method overall for each metric.

Generator	Standalone			+ HiCP2GAN			Traditional GAN-based			
	PSNR	SSIM	GenomeDISCO	PSNR	SSIM	GenomeDISCO	Method	PSNR	SSIM	GenomeDISCO
DiCARN	35.72	0.9142	0.9138*± *0.0023	**36.02$^{{\textrm{a}}}$**	**0.9213$^{{\textrm{a}}}$**	**0.9196*± *0.0012$^{{\textrm{a}}}$**	EnHiC	28.9410	0.7917	0.8484*± *0.0201
HiCARN1	35.64	0.9138	0.9106*± *0.0027	**35.93**	**0.9202**	**0.9140*± *0.002**	hicGAN	32.0452	0.8501	0.8613*± *0.0217
HiCNN	35.46	0.9085	0.9079*± *0.0048	**35.84**	**0.9161**	**0.9147*± *0.0012**	HiCARN2	35.2353	0.9128	0.9174*± *0.0026
HiCPlus	35.32	0.9051	0.9044*± *0.0034	**35.70**	**0.9124**	**0.9103*± *0.026**	DeepHiC	34.8090	0.9053	0.9105*± *0.0036

Also, as shown in Table 3, we compare the best HiCP2GAN version of the standalone generators (DiCARN) against the best traditional GAN-based model (HiCARN2) and observed that our HiCP2GAN model performed better overall. In conclusion, we observe HiCP2GAN improves all four generators on the GM12878 evaluation, supporting its use as a plug-and-play adversarial framework for same-cell Hi-C enhancement, while showing the capability to outperform the traditional GAN-based method trained from scratch.

### HiCP2GAN enables cross-cell and cross-species transfer with superior computational and biological fidelity

To assess whether HiCP2GAN’s fidelity restoration transfers beyond the training cell line, we evaluated GM12878-trained models on a held-out human cell line, K562 (Table 4), while to probe generalization beyond human data, we use a mouse Lymphoma cell line (CH12-LX) (Table 5), comparing each generator in its standalone form against its HiCP2GAN-integrated counterpart. Results for two other human cell lines, HMEC and NHEK, are reported in Supplementary Tables S10 and S11; per-chromosome values for all cross-cell evaluations are given in Supplementary Tables S5 and S6.

**Table 4:** Cross-cell evaluation on the K562 cell line. Models trained on GM12878 are evaluated on chromosomes 4, 14, 16, and 20. Bold values indicate the better result between standalone and HiCP2GAN-augmented versions of the same generator. The best traditional GAN-based method is underlined, while ($^{\textrm{a}}$) denotes the best-performing method overall for each metric. GenomeDISCO is reported as mean *± * SD across the four test chromosomes (Supplementary Table S5).

Generator	Standalone			+ HiCP2GAN			Traditional GAN-based			
	PSNR	SSIM	GenomeDISCO	PSNR	SSIM	GenomeDISCO	Method	PSNR	SSIM	GenomeDISCO
DiCARN	33.89	0.9450	0.8319*± 0.0053*	**34.53**	**0.9477$^{\textrm{a}}$**	**0.8449*± 0.0058*$^{\textrm{a}}$**	EnHiC	24.7901	0.7193	0.7558*± 0.0056*
HiCARN1	33.77	0.9319	0.8110*± 0.0050*	**34.68$^{\textrm{a}}$**	**0.9446**	**0.8316*± 0.0051***	hicGAN	31.8010	0.8107	0.7670*± 0.0116*
HiCNN	31.40	0.8535	**0.7958*± 0.0053***	**31.73**	**0.8588**	0.7060*± 0.0049*	HiCARN2	34.3449	0.9472	0.8327*± 0.0032*
HiCPlus	31.57	0.8236	0.7627*± 0.0049*	**31.81**	**0.8623**	**0.7702*± 0.0049***	HiCSR	16.1466	0.3168	0.7639*± 0.0112*

**Table 5:** Cross-cell evaluation on the CH12-LX (mouse) cell line. Models trained on GM12878 are evaluated on chromosomes 4, 14, 16, and 19. Bold values indicate the better result between standalone and HiCP2GAN-augmented versions of the same generator. The best traditional GAN-based method is underlined, while ($^{\textrm{a}}$) denotes the best-performing method overall for each metric. GenomeDISCO is reported as mean *± * SD across the four test chromosomes (Supplementary Table S6).

Generator	Standalone			+ HiCP2GAN			Traditional GAN-based			
	PSNR	SSIM	GenomeDISCO	PSNR	SSIM	GenomeDISCO	Method	PSNR	SSIM	GenomeDISCO
DiCARN	34.11	0.9666	0.6252*± 0.0740*	**35.03**	**0.9674**	**0.6402*± 0.0442***	EnHiC	26.48	0.6435	0.5505*± 0.0769*
HiCARN1	35.46	0.9700	0.6193*± 0.0743*	**35.58** $^{\textrm{a}}$	**0.9708**	**0.6984*± 0.0707*** $^{\textrm{a}}$	hicGAN	29.31	0.8854	0.5665*± 0.0746*
HiCNN	24.70	0.8254	0.5797*± 0.0668*	**29.86**	**0.8466**	**0.5812*± 0.0692***	HiCARN2	34.82	0.9720 $^{\textrm{a}}$	0.6269*± 0.0736*
HiCPlus	22.14	**0.7107**	0.5502*± 0.0748*	**22.21**	0.5106	**0.5505*± 0.0750***	HiCSR	26.88	0.4187	0.5510*± 0.0751*

Across the 16 generator-cell-line configurations spanning these four held-out cell lines, a consistent pattern emerged. PSNR improves in all 16 comparisons, and SSIM improves in 14 (the exceptions being HiCPlus on CH12-LX and HiCARN1 on HMEC). GenomeDISCO, our measure of biological reproducibility, improves in 12 comparisons, is unchanged in one (HiCNN on NHEK), and decreases in three (HiCNN on K562; HiCARN1 and HiCPlus on NHEK). The exceptions do not appear randomly distributed since two of the three GenomeDISCO decreases occur on NHEK, and degradation is most pronounced under extreme sparsity, where the 1/64 input retains too few contacts for adversarial supervision to recover fine-scale structure (Supplementary Table S7). More importantly for the framework’s central claim, the best overall GenomeDISCO in every held-out cell line is attained by a HiCP2GAN-integrated model, as indicated by the asterisks in Tables 4 and 5, and Supplementary Tables S10 and S11. HiCP2GAN also improves most pixel-level comparisons, including PSNR and SSIM, showing that the framework generally enhances local reconstruction fidelity. However, its most consistent advantage is observed in GenomeDISCO, which measures biologically reproducible contact-map structure relevant to downstream 3D genome analysis. Thus, the cross-cell results support the central design goal of HiCP2GAN: using a pretrained structure-aware discriminator to improve biologically meaningful Hi-C enhancement while maintaining strong pixel-level reconstruction performance.

All methods score lower on the mouse line than on human cells—standalone generators, HiCP2GAN variants, and the traditional GAN baselines alike, reflecting the difficulty of crossing species rather than a limitation of HiCP2GAN. GenomeDISCO on CH12-LX spans 0.55–0.70, which is lower than observed scores on K562 spanning 0.76–0.85, and varies more from chromosome to chromosome. The direction of the effect holds nonetheless, as every generator improves in PSNR and GenomeDISCO on CH12-LX, and HiCARN1 + HiCP2GAN attains the best overall values for both metrics. We therefore interpret the mouse results as evidence that the learned adversarial supervision continues to improve biological reproducibility across species, though with reduced margins and greater variance.

The comparison of these results with the same-cell results in Section HiCP2GAN improves Hi-C resolution over standalone generators and outperforms traditional GAN-based methods on seen cell line shows that a single training setup on GM12878 transfers to held-out human and mouse cell lines without cell-specific retraining. On the same-cell setting (Table 3), the HiCP2GAN-integrated DiCARN model attains the best overall PSNR, SSIM, and GenomeDISCO and remains competitive with strong conventional GAN baselines such as HiCARN2. Across the 16 held-out generator–cell-line pairings, evaluated on both computational fidelity (PSNR and SSIM) and biological reproducibility (GenomeDISCO), HiCP2GAN improves 42 of the 48 metric comparisons and attains the best overall GenomeDISCO in every cell line evaluated. The six exceptions named earlier in this section, together with the mixed results under extreme sparsity (1/64 downsampling; Supplementary Table S7), are reported explicitly. Per-chromosome GenomeDISCO values underlying these averages are reported in Supplementary Tables S5 and S6, and we describe the observed differences descriptively rather than inferentially. Overall, these results show that HiCP2GAN provides the strongest and most consistent improvement across same-cell and cross-cell evaluations, particularly for biological reproducibility, while also identifying the generator settings where gains are more limited.

### HiCP2GAN improves loop detection over standalone generators

Fine-scale chromatin loops are fundamental structural units of 3D genome organization, linking regulatory elements through long-range interactions. Accurate loop detection from enhanced Hi-C data, therefore, provides an important downstream validation of enhancement quality beyond pixel-level reconstruction metrics.

To assess biological loop recovery, we called loops from enhanced contact maps on GM12878 test chromosomes (chr4, chr14, chr16, and chr20) using Fit-Hi-C [30] and compared them with loops detected from the corresponding ground-truth 10 kb HR matrices. Recovery was quantified using the F1 score, which balances precision and recall.

Figure 3 summarizes the results. Across all evaluated generators (HiCNN, DiCARN, HiCARN1, and HiCPlus), HiCP2GAN-integrated models outperform their standalone variants, indicating that the improvements in contact-map quality translates into improved downstream structural inference. This is exemplified in HiCP2GAN-DiCARN’s performance, having a higher overall F1 score of 0.8295 compared with 0.8078 for standalone DiCARN, and this pattern is observed across the other evaluated generators, showing that the benefit of HiCP2GAN is not architecture-specific. The precision and recall scores (Supplementary Table S9) shows that HiCP2GAN increases precision and correspondingly lowers the false discovery rate for every evaluated generator (recall also increases for all but HiCARN1, which is marginally lower); the enhanced maps therefore introduce fewer loop calls unsupported by the ground truth rather than merely producing more calls, with the largest F1 gains for observed for lower-performing standalone generators.

**Figure 3:**
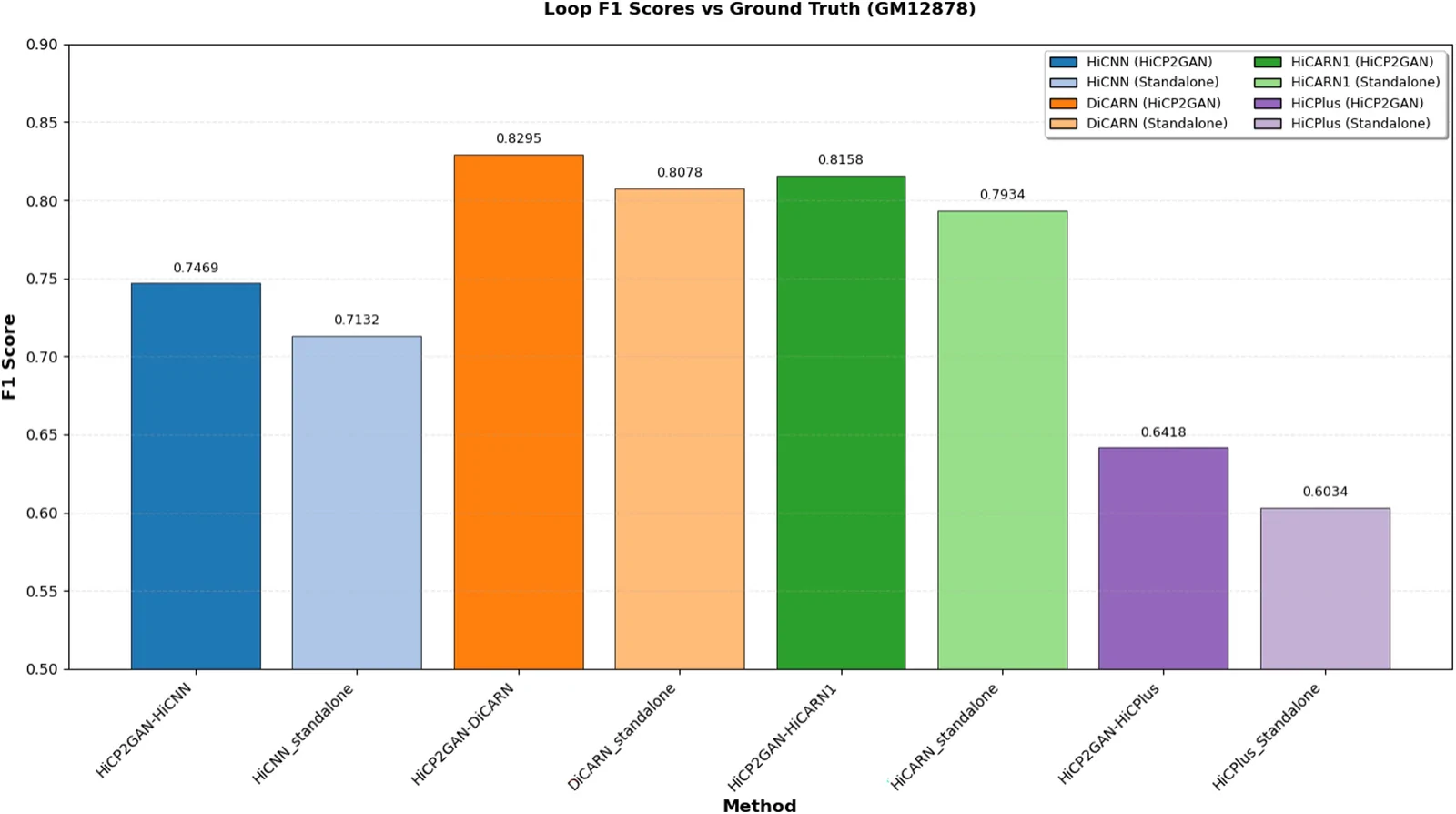
Loop F1 scores from test chromosomes (chr4, chr14, chr16, and chr20) compared with ground truth on GM12878. Dark bars represent HiCP2GAN-integrated models, and light bars represent standalone generators. Across the four evaluated generators, HiCP2GAN improves loop recovery on the held-out test chromosomes, with HiCP2GAN-DiCARN achieving the highest F1 score.

### HiCP2GAN improves topologically associating domains detection and quality

TADs are fundamental organizational units of chromatin architecture, delineating self-interaction genomic regions where contacts are significantly enriched [31]. Functions ranging enhancer-promoted facilitation to gene regulation are critically supported by this compartmentalization concept of functional genomics, especially since the disruption of TAD boundaries has been linked to anomalies in gene activation and diseases [31]. A model’s capacity to recover TADs from enhanced Hi-C data therefore serves as a biologically meaningful indicator of its utility beyond numerical reconstruction metrics.

We assessed TAD boundary recovery by applying TopDom [32] to HR and enhanced Hi-C contact maps and scoring predicted boundaries against the HR reference with the Jaccard similarity coefficient [33] (*± 5* bins, i.e. 50 kb on the 10 kb grid). Our analysis was carried out on the compact, contact-rich spans of the test chromosomes chr4 and chr14 in both GM12878 and K562, so that empty or padded intervals were excluded. For each cell line–chromosome pair, we report chromosome-scale Jaccard, and we further summarize recovery as the mean Jaccard observed across the 226 usable non-overlapping *∼ *2.4 Mb tiles spanning the same compact regions (GM12878 chr4: 78, GM12878 chr14: 36, K562 chr4: 76, K562 chr14: 36); the tile-level averages are the values reported in Table 6. Each generator was evaluated in standalone form and with HiCP2GAN under identical settings. Higher Jaccard scores indicate better boundary recovery.

**Table 6:** TAD boundary recovery across 226 non-overlapping *∼ *2.4 Mb tiles spanning the compact regions of test chromosomes chr4 and chr14 of GM12878 and K562 by mean TopDom boundary Jaccard similarity (%) against groundtruth HR references with *Δ * indicating HiCP2GAN vs. Standalone percentage points gain and bold text denoting higher recovery.

Generator	Standalone (%)	+ HiCP2GAN (%)	*Δ *
DiCARN	45.35	**45.90**	*+0.55*
HiCARN1	44.73	**47.06**	*+2.33*
HiCNN	44.70	**47.66**	*+2.96*
HiCPlus	44.57	**45.23**	*+0.66*

Table 6 reports Jaccard scores for each generator in both standalone and HiCP2GAN-based configurations, while Fig. 4 provides qualitative boundary comparisons. Across this evaluation, HiCP2GAN improves TAD boundary recovery for all four generators. The consistent direction of performance across two chromosomes of two held out cell lines indicates that the improvement is not specific to a single locus.

**Figure 4:**
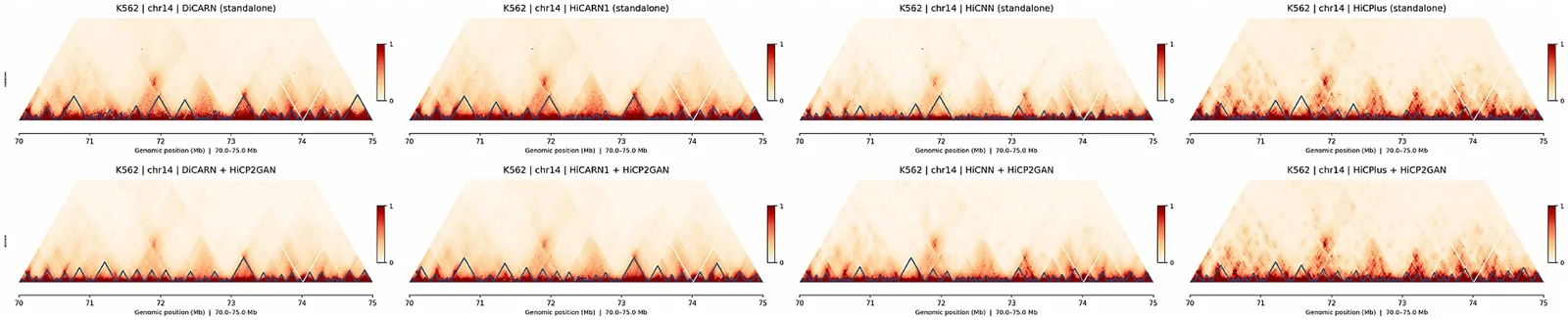
TAD contact maps for a single illustrative region, K562 chr14:70–75 Mb. Top row, standalone generators; bottom row, the corresponding HiCP2GAN variants (DiCARN, HiCARN1, HiCNN, and HiCPlus). TopDom-detected domain boundaries are marked in the figure and the quantitative concordance with the high-resolution reference across chr4 and chr14 in GM12878 and K562 is reported in Table 6.

### HiCP2GAN improves 3D genome structure reconstruction

Beyond contact-map fidelity, we evaluated whether HiCP2GAN-enhanced matrices yield more accurate local 3D genome structure reconstruction. We reconstructed the 3D structure using 3DUnicorn [34] from high-resolution (HR), standalone, and HiCP2GAN-enhanced contact maps, and scored each prediction against the HR-derived structure using the Spearman correlation coefficient (SCC) [35] of pairwise 3D distances.

To evaluate structure recovery beyond a single locus, we performed our analysis on 12 non-overlapping 400 kb windows (*40 × 40* bins at 10 kb resolution), six each from GM12878 and K562, drawn from contact-rich regions of the test chromosomes chr4, chr14, chr16, and chr20. The contact-rich window positions were selected at random and the same set of windows were used for the SCC benchmarking across all generators and configurations. Mean SCC per generator and cell line is reported in Supplementary Table S12, and the distribution across windows is shown in Fig. 5.

**Figure 5:**
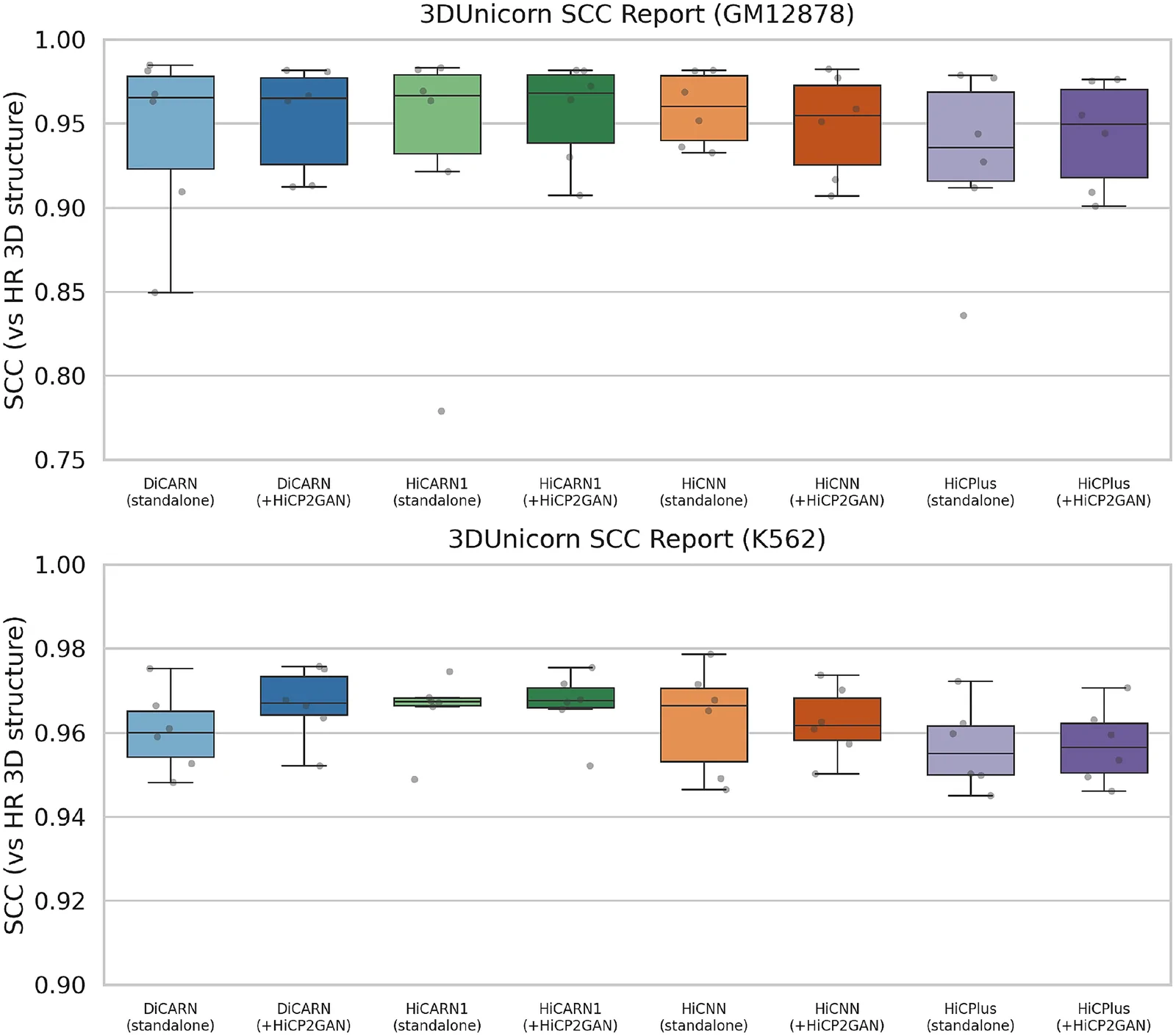
Distribution of 3DUnicorn SCC against the HR-derived reference across the evaluated 400 kb windows, for GM12878 (top) and K562 (bottom). Each generator is shown in its standalone and HiCP2GAN-integrated configuration. Points denote individual windows; boxes show the median and interquartile range.

Across the eight generator–cell-line combinations, HiCP2GAN delivers improved mean SCC in six. The gains are larger on GM12878, reaching *+0.0230* for HiCARN1, than on K562, where the differences are smaller (*≤ +0.0064*). HiCNN is the exception, where a slight decline is observed in both cell lines (*-0.0099* and *-0.0007*). Pooled across both cell lines, HiCP2GAN improves DiCARN (*+0.0083*), HiCARN1 (*+0.0121*), and HiCPlus (*+0.0074*), and reduces HiCNN (*-0.0053*).

Figure 6 shows the representative 3D structures for the GM12878 chr4:18.5–22.5 Mb region, comparing each standalone generator (top row) with its HiCP2GAN counterpart (bottom row). Supplementary Fig. S3 shows representative structure overlays for the K562 chr4:2.01–2.41 Mb window, in which the high-resolution reference, the standalone reconstruction, and the HiCP2GAN reconstruction are superimposed as ribbon models.

**Figure 6:**
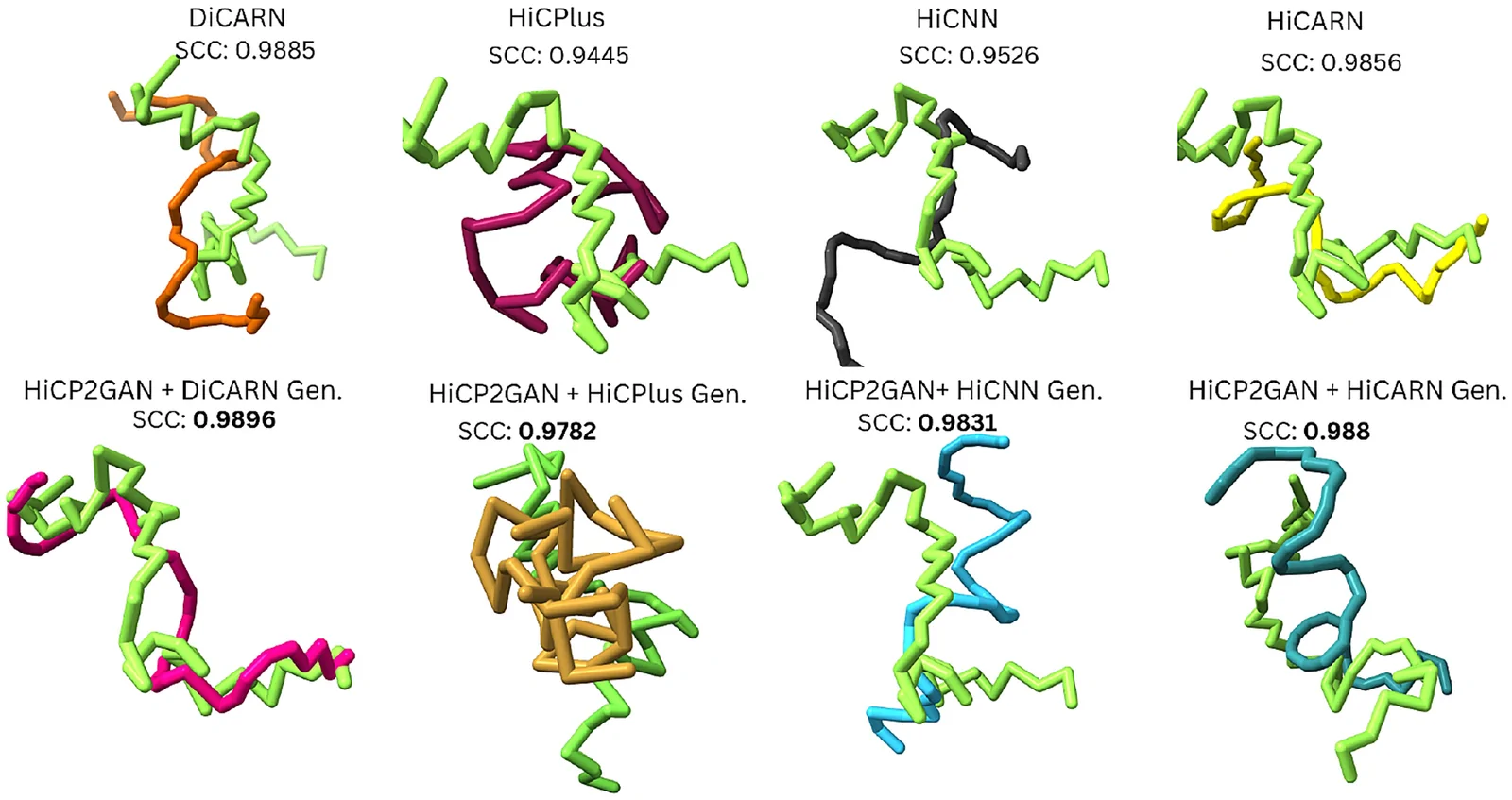
Representative 3D reconstructions of GM12878 chr4:18.5–22.5 Mb, comparing 3D structures from standalone generators predictions (top) with their HiCP2GAN counterparts (bottom), alongside the high-resolution 3D reference structure; SCC denotes Spearman correlation with the reference.

## Discussion and conclusion

HiCP2GAN introduces a plug-and-play adversarial framework for Hi-C resolution enhancement in which a transformer-based Hi-C foundation model serves as the discriminator, decoupling generator design from adversarial supervision. Our controlled ablation (Section Controlled ablation: isolating the discriminator contribution) shows that a from-scratch convolutional discriminator collapses under the same schedule, whereas transformer-based discriminators train stably; among these, the ablation clarifies the contribution of the individual components. The adversarial-only setting (l1_adv) performs comparably to reconstruction-only training (recon_only), indicating that the adversarial component alone does not account for the observed improvements. Feature matching, in contrast, provides the clearer gain. While pretrained representations improve performance further, the relatively small difference in GenomeDISCO between the randomly initialized ViT (random_vit) and the pretrained variants indicates that pretraining alone is not solely responsible for the improvement. We therefore attribute the observed gains to the feature-matching strategy, with pretrained representations providing an additional benefit. Consistent with the Phase II analysis, a compact configuration preserves this behavior without full model complexity, although the margins are smaller on a strong generator. Critically, the gains extend beyond pixel-level reconstruction: improvements in loop-detection F1 and TAD-boundary recovery indicate that this supervision extends to downstream structural inference, and the inverse relationship between standalone generator strength and HiCP2GAN gain identifies the framework as a structural floor-raiser for generators that otherwise struggle to preserve local chromatin topology.

Overall, HiCP2GAN shifts Hi-C reconstruction from architecture-specific optimization toward shared adversarial supervision and representational alignment; its generator-agnostic design and robust cross-cell generalization position it as both a benchmarking framework and a reusable training scaffold for future Hi-C enhancement methods.

**Key points**

- HiCP2GAN is a plug-and-play, generator-agnostic adversarial framework that repurposes a pretrained vision transformer-based Hi-C foundation model, trained on 118 million Hi-C patches, as the discriminator for Hi-C resolution enhancement.
- Unlike conventional Hi-C enhancement generative adversarial networks that train shallow convolutional discriminators from scratch, HiCP2GAN uses a pretrained transformer discriminator and feature-matching supervision to guide generators toward chromatin-consistent contact-map structure. Controlled ablation shows that the observed gains arise from the feature-matching strategy, with pretrained representations providing an additional benefit; a randomly initialized transformer discriminator of the same architecture also performs competitively, indicating that the adversarial component alone does not account for the improvement.
- Because the framework is generator-agnostic, any compatible enhancement architecture can serve as the generator under a single shared adversarial scaffold, enabling fair benchmarking across methods; across human cell lines it improves both pixel-level reconstruction and downstream structural inference (loop-detection F1, topologically associating domain-boundary recovery, and 3D genome structure reconstruction).

## Supplementary Material

Supplementary_HiCP2GAN____Final_Clean_bbag610

## Data Availability

HiCP2GAN is a containerized software made available via: https://github.com/OluwadareLab/HiCP2GAN. The Hi-C datasets used in this study including GM12878, K562, HMEC, NHEK, and CH12-LX [21] GEO Accession Database via GEO code GSE63525. Pretrained and trained model weights are also made available via https://zenodo.org/records/20030290.
