# Supplementary material for: HiCP2GAN: a plug and play foundation model-based GAN for Hi-C enhancement
Source: Brief Bioinform. 2026 Nov 2;27(6):bbag610. doi: 10.1093/bib/bbag610 (PMC13725921; doi:10.1093/bib/bbag610)
Supplement: Supplementary file 1 [file supplementary_hicp2gan____final_clean_bbag610.pdf]

Briefings in Bioinformatics, 2026, pp. 1–7

doi: DOI HERE

Advance Access Publication Date: Day Month Year

Supplementary Material

SUPPLEMENTARY MATERIAL

HiCP2GAN: A Plug and Play Foundation Model-based GAN for Hi-C Enhancement

Samuel Olowofila[1] and Oluwatosin Oluwadare[1,2,*]

[1]Department of Computer Science, University of Colorado at Colorado Springs, 1420 Austin Bluffs Pkwy, Colorado Springs, 80918, Colorado, USA and [2]Department of Computer Science and Engineering, University of North Texas, Denton, 76207, TX, USA

*Corresponding author. oluwatosin.oluwadare@unt.edu

Abstract

This supplementary document records implementation details, auxiliary benchmarks, and extended tables referenced from the main manuscript. Unless noted otherwise, all HiCP2GAN runs use the HiCFoundation ViT discriminator described in the main text, the generator-agnostic interface described there, and 10 kb contact maps with the chromosome-disjoint splits given in the main Methods.

1. Methods

1.a. Data sources and overlap with HiCFoundation pretraining

Table S1 lists every cell line evaluated in this study, whether it was used for HiCFoundation pretraining corpus, and the corresponding dataset accessions. All five cell lines appear in the HiCFoundation overall collected datasets: HMEC and NHEK were used for HiCFoundation pretraining, while GM12878, K562, and CH12-LX were assigned to its test split and are therefore held out from its pretraining. This information was obtained from the dataset list supplementary file from the HiCFoundation publication [2], which contains the comprehensive accession list for all the datasets used in their study. All Hi-C data used for training and evaluation in the present work are from Rao et al. [1] (GEO accession GSE63525).

1.b. Architecture overview

Figure S1 summarizes the data flow and losses used in HiCP2GAN; it extends the conceptual overview in the main manuscript with implementation-level detail.

1.c. Default training hyperparameters

Table S2 lists the default training and optimization settings used for the reported HiCP2GAN experiments, except where a hyperparameter study explicitly varies a quantity (e.g. discriminator depth in the freeze sweep).

1.d. Discriminator adaptation (HiCFoundation)

In section 3.2 of the manuscript, we describe a compact operating point with the first eleven transformer blocks finetuned and deeper blocks frozen (Phase II), following the validation study summarized there. Feature matching applies ℓ_1 loss to the discriminator embedding used for the adversarial head (the same tensor as $\phi(\cdot)$ in the main text).

1.e. Generator integration

Resolution adaptation ($40 \rightarrow 28 \rightarrow 40$). HiCSR, HiCNN, and HiCPlus are wrapped so that LR patches are downsampled with area interpolation to 28×28, the native generator runs at that resolution, and outputs are upsampled to 40×40 with nearest-neighbour interpolation. HiCARN1, DiCARN, and DeepHiC consume 40×40 patches directly.

Output normalization. HiCSR tanh outputs are mapped to $[0, 1]$ via $(x+1)/2$; all generator outputs and HR targets are clamped to $[0, 1]$ before reconstruction and GenomeDISCO computations.

1.f. Low-resolution downsampling protocol (1/16 vs. 1/64)

Following Rao et al. [1] high-depth maps, low-resolution inputs are generated by uniform downsampling of contact counts to 1/16 or 1/64 of effective sequencing depth, applied consistently across train, validation, and test partitions. Primary manuscript metrics for same-cell GM12878 and cross-cell transfer use 1/16 downsampling unless a supplementary table explicitly reports 1/64.

© The Author 2026. Published by Oxford University Press. All rights reserved. For permissions, please e-mail: journals.permissions@oup.com

Table S1. Data sources for the cell lines evaluated in this study and their inclusion status in the HiCFoundation pretraining corpus. "Yes" indicates that the cell line was included in HiCFoundation pretraining. "Held out from HiCP2GAN" column indicates that the cell line was not used in our task-specific adversarial training, which used GM12878 only. "Our Study Accession" column gives the GEO accession number for the dataset analyzed in this study, whereas "HiCFoundation Dataset Accession(s)" column lists the accession numbers of the corresponding cell-line datasets collected for the development and evaluation of HiCFoundation.

Cell line	Role in our Study	In HiCFoundation Pretraining Corpus	Held out from HiCP2GAN	Our study Accession	HiCFoundation Dataset Accession(s)
GM12878	Train + same-cell test	No	No	GSE63525	4DNES3JX38V5, 4DNES4269GKX, 4DNESCIHJOXA, 4DNESJFTAURO, 4DNESLLA3R1V, 4DNESLQG7ZKJ, 4DNESPXW8XHY, 4DNESZMKWAD3, 4DNESLLTENG9, 4DNESNZZR2VD, 4DNESQWI9K2F, 4DNESBJ1KYYH, 4DNESNDJ9QVX, ENCSR410MDC, ENCSR730CER, ENCSR916MFV, ENCSR742SAT, ENCSR968KAY, ENCSR382RFU, ENCSR046XXF, ENCSR423SYP
K562	Cross-cell test	No	Yes	GSE63525	4DNESI7DEJTM, 4DNESPOC41XG, 4DNESU95RUNO, 4DNES1E3ET5M, 4DNES9J6QJQS, 4DNESQICH2XW, 4DNESR93N4E3, 4DNESVZ6QH33, 4DNES54GS5KI, 4DNESC23ZYOF, 4DNESCQRIZ7D, 4DNESF17LNZE, 4DNESFM66XDL, 4DNESHJVC7MP, 4DNESLVTLWPX, 4DNESMG7JML8, 4DNESPXSO8GB, 4DNESQV6Y4JL, 4DNESSH9ICEW, 4DNEST7Y7S69, 4DNESYVC49SD, 4DNESZUWCRVN, ENCSR545YBD, ENCSR479XDG
CH12-LX	Cross-species test	No	Yes	GSE63525	4DNES4GDH4BG, 4DNES7C6LBWI, 4DNESK95HVFB, 4DNES6YMW2WC, ENCSR005UON, ENCSR549VZT, ENCSR338LIB
HMEC	Cross-cell test	Yes	Yes	GSE63525	ENCSR380YQA, ENCSR711AVS, ENCSR707XVJ, ENCSR080ODG
NHEK	Cross-cell test	Yes	Yes	GSE63525	4DNESECNR4O8, 4DNESYPKLMAM

Table S2. Core training and optimization settings for HiCP2GAN experiments.

**Setting**	**Value**
Patch size (LR / HR)	40 × 40 bins
Batch size	64
Training epochs (main experiments)	100
Mixed precision (AMP)	Enabled
Optimizer	Adam, β_1=0.5, β_2=0.999, weight decay 0
Learning rate — generator	2×10^{-4}
Learning rate — discriminator	2×10^{-4}
$\lambda_{\text{adv}}, \lambda_{L1}, \lambda_{\text{FM}}$	1, 10, 10
Gradient clipping (max norm)	1.0 (generator and discriminator)
Random seed	1337
ViT backbone	ViT-L/16 (HiCFoundation)
Discriminator MLP width	256

1.g. Hyperparameter search

1.h. Extended discriminator training (500 epochs)

The main text cites this table when noting that 500-epoch training of the top Phase I configurations yields only marginal PSNR gains relative to the 100-epoch protocol. HiCARN1 served as the generator; metrics are mean validation PSNR on GM12878.

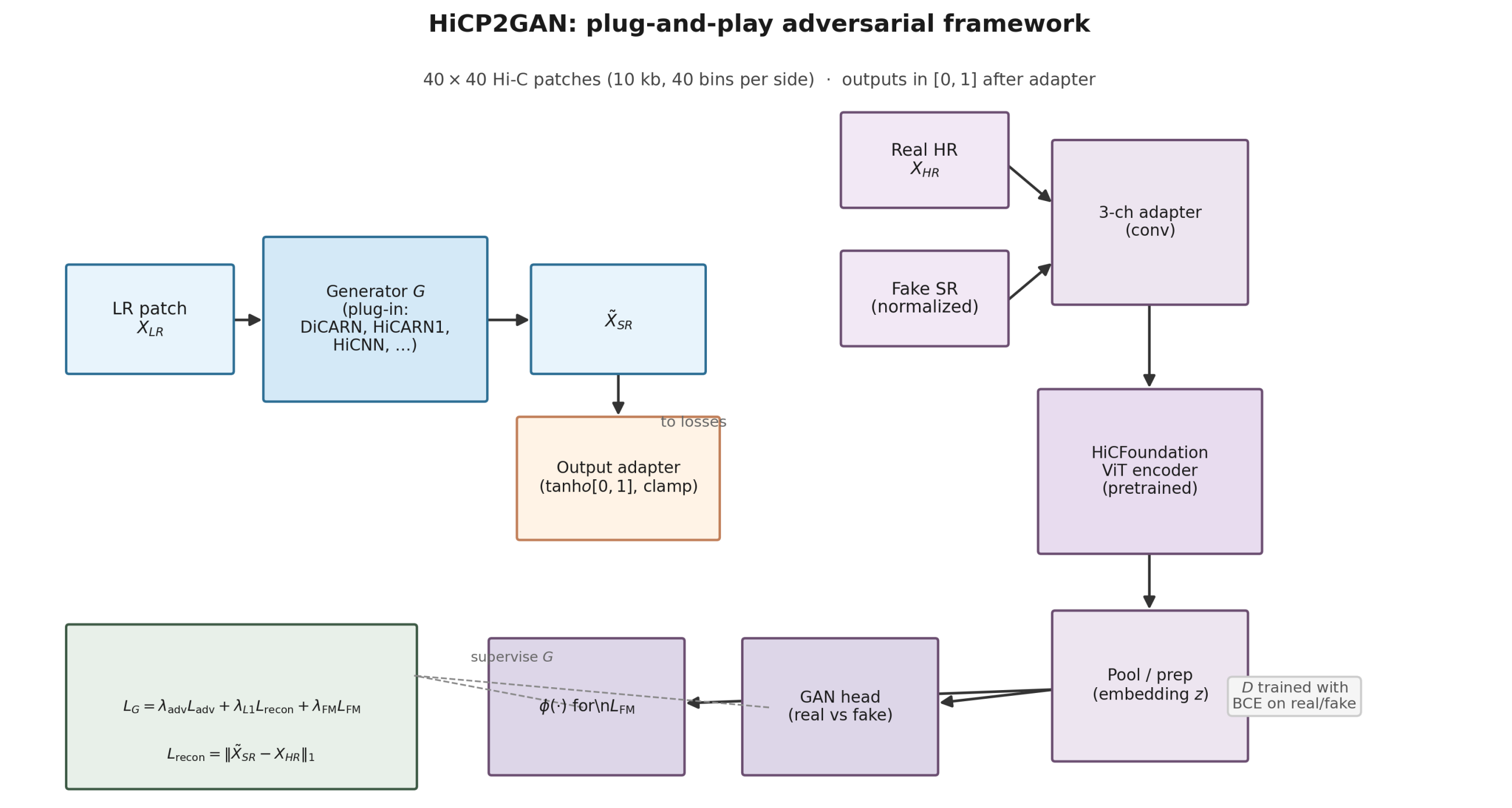

Fig. S1. Overview of HiCP2GAN. Left: a plug-in generator G maps a low-resolution patch X_{LR} to $\tilde{X}_{SR}$, followed by the module-aware output adapter involving tanh rescaling and clamping to $[0, 1]$. Right: the discriminator processes real X_{HR} and normalized fake $\tilde{X}_{SR}$ through a three-channel convolutional adapter and the HiCFoundation ViT encoder; pooled embeddings feed both the adversarial head (BCE) and the representation $\phi(\cdot)$ used in feature matching. Bottom: the composite generator objective combines adversarial, ℓ_1 reconstruction, and feature-matching terms; the discriminator is trained alternately on real and fake inputs.

Table S3. Phase I discriminator finetuning-depth sweep on GM12878 (100 epochs). Average validation PSNR when the first N transformer blocks of the HiCFoundation backbone are finetuned and the remaining $24-N$ deeper blocks are frozen. HiCARN1 is used as the generator throughout.

Finetuned	PSNR	Finetuned	PSNR	Finetuned	PSNR
0	35.92	9	35.92	18	35.92
1	**35.93**	10	**35.93**	19	35.91
2	35.92	11	35.92	20	35.91
3	35.92	12	35.91	21	35.91
4	35.91	13	**35.93**	22	35.92
5	35.92	14	35.92	23	35.92
6	35.92	15	35.92	24	35.91
7	35.92	16	**35.93**		
8	35.92	17	**35.93**		

Table S4. Extended 500-epoch training for selected discriminator finetuning depths. Average validation PSNR (dB) on GM12878 with HiCARN1 as the generator.

Finetuned transformer blocks (from input)	PSNR (dB)
10	35.93
13	**35.94**
16	35.93
17	35.93

2. Results

2.a. Per-chromosome cross-cell metrics (1/16)

Table S5 lists PSNR (dB), SSIM, and GenomeDISCO on each held-out test chromosome (chr4, chr14, chr16, chr20) for models trained on GM12878 and evaluated on HMEC, K562, and NHEK (1/16 downsampling). Pooled metrics appear in the main text; this table gives the per-chromosome breakdown with cross-chromosome variation. The GenomeDISCO mean $\pm$ SD values reported in

main-text Tables 4–6 are the sample mean and sample standard deviation of these four chromosome scores; paired chromosome-level t-tests are given in Supplementary Table S7.

Table S5: Per-chromosome cross-cell evaluation (1/16 downsampling) on test chromosomes 4, 14, 16, and 20. *Note: All values are compressed to 4 decimal points.*

			**Standalone**			**+ HiCP2GAN**		
**Cell line**	**Chr**	**Generator**	PSNR	SSIM	GDISCO	PSNR	SSIM	GDISCO
HMEC (cross-cell, 1/16 LR)								
HMEC	chr4	DiCARN	31.92	0.8571	0.5102	33.47	0.8823	0.5145
HMEC	chr4	HiCARN1	33.21	0.8765	0.4958	33.42	0.8665	0.5748
HMEC	chr4	HiCNN	26.11	0.8545	0.4942	27.28	0.8741	0.4961
HMEC	chr4	HiCPlus	25.67	0.7523	0.4380	28.88	0.7845	0.4548
HMEC	chr14	DiCARN	32.44	0.8612	0.5156	33.89	0.8851	0.5199
HMEC	chr14	HiCARN1	33.78	0.8798	0.5012	33.91	0.8698	0.5806
HMEC	chr14	HiCNN	26.45	0.8589	0.4998	27.67	0.8782	0.5018
HMEC	chr14	HiCPlus	25.98	0.7567	0.4432	29.15	0.7889	0.4599
HMEC	chr16	DiCARN	33.18	0.8645	0.5211	34.56	0.8889	0.5254
HMEC	chr16	HiCARN1	34.44	0.8834	0.5068	34.38	0.8723	0.5863
HMEC	chr16	HiCNN	27.02	0.8618	0.5053	28.34	0.8809	0.5072
HMEC	chr16	HiCPlus	26.34	0.7589	0.4483	29.67	0.7912	0.4649
HMEC	chr20	DiCARN	32.78	0.8684	0.5214	34.56	0.8913	0.5258
HMEC	chr20	HiCARN1	34.53	0.8867	0.5070	34.65	0.8758	0.5859
HMEC	chr20	HiCNN	27.06	0.8656	0.5051	28.39	0.8844	0.5070
HMEC	chr20	HiCPlus	26.45	0.7629	0.4485	29.94	0.7942	0.4650
K562 (cross-cell, 1/16 LR)								
K562	chr4	DiCARN	33.22	0.9412	0.8251	33.87	0.9440	0.8389
K562	chr4	HiCARN1	33.11	0.9281	0.8048	34.02	0.9410	0.8254
K562	chr4	HiCNN	30.89	0.8498	0.7892	31.22	0.8550	0.7002
K562	chr4	HiCPlus	31.08	0.8198	0.7565	31.33	0.8585	0.7640
K562	chr14	DiCARN	33.65	0.9438	0.8302	34.23	0.9465	0.8425
K562	chr14	HiCARN1	33.54	0.9307	0.8095	34.44	0.9435	0.8301
K562	chr14	HiCNN	31.23	0.8523	0.7943	31.54	0.8576	0.7049
K562	chr14	HiCPlus	31.42	0.8224	0.7612	31.66	0.8611	0.7688
K562	chr16	DiCARN	34.21	0.9465	0.8343	34.89	0.9492	0.8463
K562	chr16	HiCARN1	34.08	0.9335	0.8132	35.01	0.9462	0.8336
K562	chr16	HiCNN	31.67	0.8549	0.7984	31.99	0.8601	0.7080
K562	chr16	HiCPlus	31.84	0.8248	0.7658	32.07	0.8637	0.7729
K562	chr20	DiCARN	34.48	0.9485	0.8380	35.13	0.9511	0.8519
K562	chr20	HiCARN1	34.35	0.9353	0.8165	35.25	0.9487	0.8373
K562	chr20	HiCNN	31.81	0.8570	0.8013	32.17	0.8625	0.7109
K562	chr20	HiCPlus	31.94	0.8274	0.7673	32.18	0.8660	0.7751
NHEK (cross-cell, 1/16 LR)								
NHEK	chr4	DiCARN	34.78	0.9611	0.8431	35.02	0.9632	0.8485
NHEK	chr4	HiCARN1	34.72	0.9518	0.8146	35.01	0.9657	0.8101
NHEK	chr4	HiCNN	33.68	0.8805	0.8116	34.67	0.9066	0.8116
NHEK	chr4	HiCPlus	32.71	0.8468	0.8322	33.57	0.8665	0.7925
NHEK	chr14	DiCARN	35.11	0.9628	0.8478	35.33	0.9648	0.8532
NHEK	chr14	HiCARN1	35.07	0.9534	0.8194	35.31	0.9674	0.8150
NHEK	chr14	HiCNN	33.92	0.8821	0.8165	34.91	0.9082	0.8165
NHEK	chr14	HiCPlus	33.02	0.8484	0.8371	33.89	0.8682	0.7974
NHEK	chr16	DiCARN	35.48	0.9646	0.8510	35.71	0.9666	0.8564
NHEK	chr16	HiCARN1	35.44	0.9550	0.8228	35.68	0.9692	0.8182
NHEK	chr16	HiCNN	34.23	0.8837	0.8198	35.29	0.9098	0.8198
NHEK	chr16	HiCPlus	33.28	0.8499	0.8402	34.15	0.8698	0.8002
NHEK	chr20	DiCARN	35.95	0.9659	0.8553	36.26	0.9674	0.8607
NHEK	chr20	HiCARN1	35.89	0.9562	0.8264	36.20	0.9701	0.8219
NHEK	chr20	HiCNN	34.69	0.8857	0.8233	35.65	0.9118	0.8233
NHEK	chr20	HiCPlus	33.59	0.8513	0.8441	34.47	0.8711	0.8047

Table S6: Per-chromosome cross-species evaluation (1/16 downsampling) on test chromosomes 4, 14, 16, and 19 for CH12-LX.

			**Standalone**			**+ HiCP2GAN**		
**Cell line**	**Chr**	**Generator**	PSNR	SSIM	GDISCO	PSNR	SSIM	GDISCO
CH12-LX (cross-cell, 1/16 *LR)*								
CH12-LX	chr4	DiCARN	34.04	0.9587	0.5424	34.97	0.9606	0.5905
CH12-LX	chr4	HiCARN1	35.41	0.9644	0.5378	35.53	0.9652	0.6198
CH12-LX	chr4	HiCNN	24.61	0.8007	0.5051	29.79	0.8249	0.5050
CH12-LX	chr4	HiCPlus	22.05	0.6822	0.4668	22.12	0.4750	0.4670
CH12-LX	chr14	DiCARN	34.09	0.9659	0.6156	35.01	0.9668	0.6346
CH12-LX	chr14	HiCARN1	35.44	0.9695	0.6104	35.56	0.9703	0.6915
CH12-LX	chr14	HiCNN	24.68	0.8228	0.5713	29.84	0.8441	0.5728
CH12-LX	chr14	HiCPlus	22.12	0.7076	0.5404	22.19	0.5045	0.5407
CH12-LX	chr16	DiCARN	34.13	0.9681	0.6331	35.05	0.9689	0.6488
CH12-LX	chr16	HiCARN1	35.48	0.9712	0.6277	35.60	0.9720	0.7064
CH12-LX	chr16	HiCNN	24.73	0.8279	0.5886	29.88	0.8492	0.5897
CH12-LX	chr16	HiCPlus	22.16	0.7135	0.5584	22.23	0.5101	0.5587
CH12-LX	chr19	DiCARN	34.18	0.9737	0.7097	35.09	0.9733	0.6869
CH12-LX	chr19	HiCARN1	35.51	0.9749	0.7013	35.63	0.9757	0.7759
CH12-LX	chr19	HiCNN	24.78	0.8502	0.6538	29.93	0.8682	0.6573
CH12-LX	chr19	HiCPlus	22.23	0.7395	0.6352	22.30	0.5528	0.6356

2.b. 1/16 vs. 1/64 low-resolution depth (GM12878)

The 1/16 columns repeat the chromosome-pooled test metrics from the main manuscript (same-cell GM12878 evaluation on chromosomes 4, 14, 16, and 20). The 1/64 columns report the same metrics computed on the 1/64 corpus (test chromosomes).

Table S7. GM12878 contact-map metrics under 1/16 vs. 1/64 downsampling (test chromosomes 4, 14, 16, 20).

	1/16 **LR (test)**			1/64 **LR (test)**		
**Setting**	PSNR	SSIM	GDISCO	PSNR	SSIM	GDISCO
DiCARN standalone	35.72	0.9142	0.9138	32.84	0.8754	0.8795
DiCARN + HiCP2GAN	36.02	0.9213	0.9196	33.35	0.8886	0.8756
HiCARN1 standalone	35.64	0.9138	0.9106	33.61	0.8900	0.8905
HiCARN1 + HiCP2GAN	35.93	0.9202	0.9140	33.25	0.8873	0.8796
HiCNN standalone	35.46	0.9085	0.9079	28.01	0.3822	0.8342
HiCNN + HiCP2GAN	35.84	0.9161	0.9147	28.31	0.3822	0.8418
HiCPlus standalone	35.32	0.9051	0.9044	26.63	0.5650	0.8324
HiCPlus + HiCP2GAN	35.70	0.9124	0.9103	29.11	0.5812	0.8292

2.c. Controlled discriminator/loss ablation

To isolate the contribution of the pretrained discriminator from discriminator architecture, capacity, and the auxiliary loss terms, we hold the generator (HiCARN1), optimization schedule, training data, and evaluation fixed, and vary only the discriminator or the active loss terms. Metrics are validation results on GM12878 under 1/16 downsampling (100 epochs). GenomeDISCO is reported as mean $\pm$ SD across the evaluation chromosomes. On this strong generator the pixel-fidelity metrics (PSNR, SSIM) are near saturation, so the discriminating signal is GenomeDISCO. The proposed partially finetuned HiCFoundation discriminator (HiCP2GAN) attains the best score on every metric. The pretrained discriminators (partial-FT and frozen) exceed the randomly initialized ViT on GenomeDISCO, indicating that the pretrained representations; not the transformer architecture or capacity alone, supply the biologically useful supervision; feature matching is the operative loss term (adding it raises GenomeDISCO from 0.909 to 0.913, whereas the adversarial term alone matches the reconstruction-only baseline). The conventional from-scratch CNN discriminator collapsed, far underperforming all transformer variants. Training wall-clock times are reported for the same hardware protocol. Abbreviations interprets

2.d. Extended biological validation: loop precision/recall/FDR and CTCF enrichment

To characterize loop recovery beyond the F1 summary in Figure 3, we decompose the same Fit-Hi-C loop calls on the GM12878 test chromosomes (chr4, 14, 16, and 20) into precision, recall, and false discovery rate (FDR = 1−precision); the F1 column reproduces Figure 3 (Table S9). For every generator, HiCP2GAN increases precision and lowers FDR; recall also increases for three of the four generators and is marginally lower only for HiCARN1 (0.794 → 0.780). The increase in precision (equivalently, the reduction in FDR) across all generators means the enhanced maps yield fewer loop calls unsupported by the ground truth, directly addressing the concern that a generative model may introduce false-positive contacts, while overall F1 improves for every generator. The largest F1 gains are observed for the weaker generators (HiCNN and HiCPlus).

Table S8. Controlled discriminator and loss ablation on GM12878 (validation, 1/16 downsampling, HiCARN1 generator). FM = feature matching; Adv = adversarial loss; FT = finetuned. The conventional CNN discriminator was unstable (losses diverged to NaN); its best early-checkpoint values are shown for reference. Best result per metric in bold. GenomeDISCO is reported as mean ± SD across the evaluation chromosomes.

Setting	Discriminator	Pretrained	PSNR	SSIM	GenomeDISCO	Train time
recon_only	none	–	35.90	0.924	0.909 ± 0.0101	8h 14m
cnn_disc	CNN (from scratch)	No	32.25	0.881	0.891 ± 0.0027	8h 54m
random_vit	ViT (random init)	No	35.84	0.924	0.912 ± 0.0033	12h 16m
l1_adv	HiCFoundation FT	Yes	35.89	0.924	0.909 ± 0.0104	12h 12m
l1_fm	HiCFoundation FT	Yes	35.91	0.925	0.913 ± 0.0041	12h 07m
frozen_pretrained	HiCFoundation frozen	Yes	35.92	0.925	0.914 ± 0.0027	10h 36m
partial_ft (HiCP2GAN)	HiCFoundation FT	Yes	**35.93**	**0.925**	**0.915 ± 0.0021**	11h 09m

Table S9. Loop recovery on GM12878 test chromosomes (chr4, 14, 16, 20), decomposing the F1 scores of Figure 3 into precision (P), recall (R), and false discovery rate (FDR = 1−P). Loops were called with Fit-Hi-C and matched to loops from the ground-truth 10 kb high-resolution maps. Bold marks the better standalone-vs-HiCP2GAN value per generator.

Generator	Configuration	Precision	Recall	FDR	F1
DiCARN	standalone	0.845	0.774	0.155	0.8078
DiCARN	+ HiCP2GAN	**0.870**	**0.793**	**0.130**	**0.8295**
HiCARN1	standalone	0.793	**0.794**	0.207	0.7934
HiCARN1	+ HiCP2GAN	**0.855**	0.780	**0.145**	**0.8158**
HiCNN	standalone	0.750	0.680	0.250	0.7132
HiCNN	+ HiCP2GAN	**0.785**	**0.712**	**0.215**	**0.7469**
HiCPlus	standalone	0.603	0.604	0.397	0.6034
HiCPlus	+ HiCP2GAN	**0.680**	**0.608**	**0.320**	**0.6418**

To assess biological support independent of the Hi-C maps, we tested whether insulation-based TAD boundaries recovered from enhanced K562 maps are enriched for CTCF, using ENCODE K562 CTCF optimal IDR peaks (ENCFF002CEL, hg19) against a region-matched genomic null (200 permutations). Recovered boundaries (±20 kb) are CTCF-enriched relative to null (typically 1.5–1.8-fold), approaching the ground-truth enrichment (mean ≈ 1.75). Mean TAD-boundary enrichment is modestly higher for HiCP2GAN than for the standalone generator (1.65 vs. 1.58 for DiCARN); we do not formally test this difference and therefore do not claim CTCF superiority. TAD midpoints show no enrichment (negative control), as expected, and loop-anchor CTCF enrichment under the permissive caller was near null and is not interpreted as support. This analysis is limited to K562 and to the generators (DiCARN, HiCARN1) whose reconstructions yielded stable insulation calls.

Table S10. Cross-cell evaluation on the HMEC cell line. Models trained on GM12878 are evaluated on chromosomes 4, 14, 16, and 20. Bold values indicate the better result between standalone and HiCP2GAN-augmented versions of the same generator. The best traditional GAN-based method is underlined, while (*) denotes the best-performing method overall for each metric. GenomeDISCO is reported as mean ± SD across the four test chromosomes (Supplementary Table S4).

Generator	Standalone			+ HiCP2GAN			Traditional GAN-based			
	PSNR	SSIM	GenomeDISCO	PSNR	SSIM	GenomeDISCO	Method	PSNR	SSIM	GenomeDISCO
DiCARN	32.58	0.8628	0.5171±0.0051	**34.12**	**0.8869**	**0.5214**±0.0053	EnHiC	26.76	0.6416	0.4425±0.0064
HiCARN1	33.99	**0.8816**	0.5027±0.0053	**34.09**	0.8711	**0.5819**±0.0054*	hicGAN	31.13	0.8807	0.4371±0.0026
HiCNN	26.66	0.8602	0.5011±0.0053	**27.92**	**0.8794**	**0.5030**±0.0053	HiCARN2	<u>35.06*</u>	<u>0.9741*</u>	<u>0.5185±0.0096</u>
HiCPlus	26.11	0.7577	0.4445±0.0050	**29.41**	**0.7897**	**0.4612**±0.0049	HiCSR	16.07	0.3971	0.4448±0.0109

Table S11. Cross-cell evaluation on the NHEK cell line. Models trained on GM12878 are evaluated on chromosomes 4, 14, 16, and 20. Bold values indicate the better result between standalone and HiCP2GAN-augmented versions of the same generator. The best traditional GAN-based method is underlined, while (*) denotes the best-performing method overall for each metric. GenomeDISCO is reported as mean ± SD across the four test chromosomes (Supplementary Table S4).

Generator	Standalone			+ HiCP2GAN			Traditional GAN-based			
	PSNR	SSIM	GenomeDISCO	PSNR	SSIM	GenomeDISCO	Method	PSNR	SSIM	GenomeDISCO
DiCARN	35.33	0.9636	0.8493±0.0052	**35.58***	**0.9655**	**0.8547**±0.0052*	EnHiC	25.39	0.6589	0.5662±0.0067
HiCARN1	35.28	0.9541	**0.8208**±0.0050	**35.55**	**0.9681**	0.8163±0.0050	hicGAN	31.39	0.8250	0.5743±0.0088
HiCNN	34.13	0.8830	0.8178±0.0050	**35.13**	**0.9091**	**0.8178**±0.0050	HiCARN2	<u>35.08</u>	<u>0.9712*</u>	<u>0.6497±0.0053</u>
HiCPlus	33.15	0.8491	**0.8384**±0.0050	**34.02**	**0.8689**	0.7987±0.0051	HiCSR	16.08	0.255	0.5689±0.0069

Table S12. Mean SCC of 3DUnicorn structures versus the HR reference over 12 400 kb contact-rich windows from held-out chromosomes chr4, chr14, chr16, and chr20. Values are averaged over windows; Δ is HiCP2GAN − standalone.

Cell	Generator	HiCP2GAN	Standalone	Δ
GM12878	DiCARN	0.9532	0.9429	+0.0103
GM12878	HiCARN1	0.9563	0.9333	+0.0230
GM12878	HiCNN	0.9490	0.9590	−0.0099
GM12878	HiCPlus	0.9436	0.9293	+0.0144
K562	DiCARN	0.9668	0.9605	+0.0064
K562	HiCARN1	0.9667	0.9655	+0.0012
K562	HiCNN	0.9625	0.9632	−0.0007
K562	HiCPlus	0.9571	0.9566	+0.0005
Overall	DiCARN	0.9600	0.9517	+0.0083
Overall	HiCARN1	0.9615	0.9494	+0.0121
Overall	HiCNN	0.9558	0.9611	−0.0053
Overall	HiCPlus	0.9504	0.9429	+0.0074

2.f Representative 3D structure overlays (K562)

Figure S2 shows representative 3D structure overlays for the K562 chr4:2.01–2.41 Mb window, complementing the GM12878 reconstructions in Figure 6 of the main manuscript. The per-window SCC values across all evaluated windows are reported in Table S12.

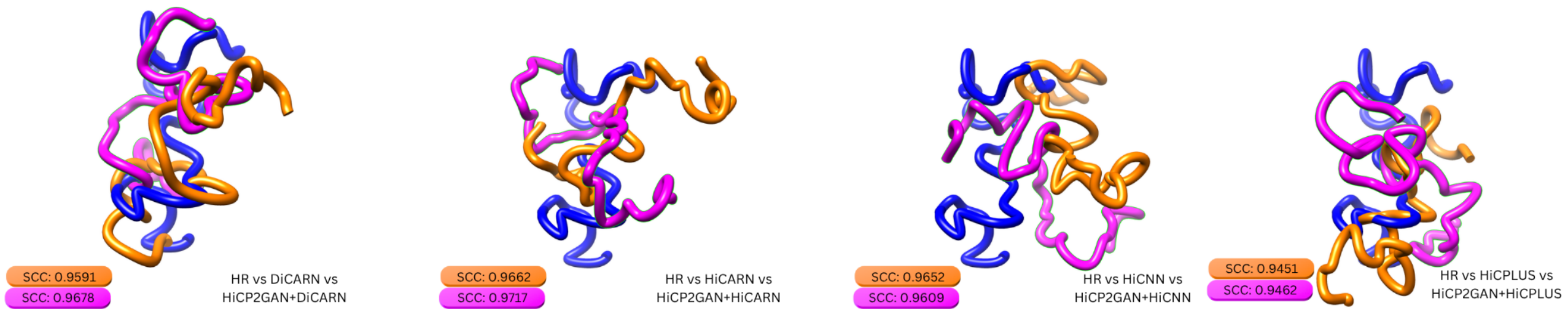

Fig. S2. Representative 3D structure overlays for K562 chr4:2.01–2.41 Mb, shown as ribbon models. Each panel superimposes the high-resolution reference (blue) with the standalone reconstruction (orange) and the corresponding HiCP2GAN reconstruction (magenta), for DiCARN, HiCARN1, HiCNN, and HiCPlus. Labels report the Spearman correlation coefficient (SCC) of pairwise distances against the reference: orange for the standalone model, magenta for the HiCP2GAN variant.

References

1. Rao SS, Huntley MH, Durand NC, Stamenova EK, Bochkov ID, Robinson JT, Sanborn AL, Machol I, Omer AD, Lander ES, et al. A 3d map of the human genome at kilobase resolution reveals principles of chromatin looping. Cell, 159(7):1665–1680, 2014.
2. Wang X, Zhang Y, Ray S, Jha A, Fang T, Hang S, Doulatov S, Noble WS, and Wang S. A generalizable hi-c foundation model for chromatin architecture, single-cell and multiomics analysis across species. Nature Methods, 23(7):1334–1348, 2026.
